# Food Products and Digital Tools: The Unexpected Interconnections

**DOI:** 10.3389/fnut.2022.847038

**Published:** 2022-02-17

**Authors:** Francesco Marra

**Affiliations:** Virprofood, Department of Industrial Engineering, University of Salerno, Fisciano, Italy

**Keywords:** food product design, digital tools, food ingredients, food process modeling, food and social networks

## Abstract

This article discusses the current advances and proposes future directions in the use of science-based digital tools in food product design, highlighting some unexpected interconnections among tools science-based and tools thought for other purposes. The article is structured in two main parts: an overview of the literature on the work done to explore food-related apps and social media for understanding consumers' perception and preferences; a discussion on the integration of consumers' perception and preferences in a wider scheme for food product design based on a prediction of product features using advanced multiscale and hybrid methods for the design of food product features associated to consumer perception and preferences. Understanding consumer needs and preferences and linking them to product features will benefit start-uppers and researchers who develop tools for reinventing food product design.

## Introduction

A portion of food is a formulated product, a mix of ingredients (sometimes, many ingredients), undergoing a sequence of manipulation processes (sometimes, many processes). The design of food products involves – on one side – the capability to capture the consumers' needs and preferences, and – on the other side – the capabilities to translate these preferences in product attributes related to the dichotomy of ingredients' choice and manipulation. Thus, the design procedure requires three steps: (1) identification of the consumer needs and preferences; (2) translation of these needs and preferences into chemical/physical properties and features; (3) combination of ingredients in a mix and in several operations (what we call process) to obtain a product that is characterized by these properties and features.

In the design of new food products, an important role is played by the consumers' needs and preferences since they determine the final characteristics that a food product must have so that the consumer will select this product above the others. Consumer needs and preferences can deal with different aspects, some of them being:

- the food product price (yes, price is still one of the most important tenets when choosing food);
- the food product taste (besides any possible health claim, some consumers are just attracted by the sensorial experience a certain food product will bring to them);
- the food product composition (a vegan consumer will not buy a product made with animal-based ingredients; a consumer guided by healthy motivation will avoid consuming food high in salt, fat or sugar).

Certainly, other aspects should be taken into account, such as the supply chain and the logistics, the consumption trends, the sustainability.

Big food companies knew and know how to perform marketing research, they have their experts in understanding the consumers' trends and their experts in food product formulation and food process design. Small and medium food companies (often still run as family companies) own their traditional recipe and can exploit their competence in some market niches. This leads to the question, why should they explore new ways, digitally based, to improve their food design capabilities?

Digital tools, undoubtedly, allow shortening the time-to-market (1). Being based on computation, they can virtually provide answers to complex problems in a short time, exploring scenarios with a very limited cost and – potentially – without any limit. So, in a world running toward personalized nutrition, the possibility to tremendously speed up the time-to-market would open new roads to established food companies but also to start-ups (2).

From an engineering point of view, the design of a food problem is a multi-scale, interdisciplinary problem, its heart being the optimization of different product specifications. As in a classical pooling problem (3), the goal is to find the lowest cost flow rates in the network that satisfy the market demands. Talking about cost, one should also consider sustainability, including environmental and social costs. It is possible to use mathematical methods, software, and any other possible virtual/digital tool to fulfill all the relationships (input-toward-output, so consumers' need and preferences toward final product characteristics, along important intermediate steps related to ingredients' choice and process choice) and thus to design a new product (or to optimize an existing one).

In the following discussion, the logistics and the supply chain are not considered, since there are consolidated methodologies and software instruments to comply with it (4), and the digital tools that can be used for determining the needs and preferences of customers are first introduced and discussed; subsequently, the discussion will deal with the possibility to combine these tools with other methodologies (standardized and not) for predicting the effect of processing on a food product, for optimizing the ingredient choice and the recipe.

For further discussion on selected approaches, best practices, hurdles, and limitations regarding knowledge transfer *via* software and the mathematical models embedded in it, the work recently published by Kansou et al. (5) can establish a reference point for the food community.

## New Ways to Identify and Translate the Consumer Needs

As in other industrial sectors, also in the food industry, the design of a product according to the consumers' needs is surely a key target (6). Customers' needs, and desires would determine the willingness of consumers to pay for a particular product. The biggest challenge is then represented by the ability of a food producer to understand the consumers' needs, capture their attention, and transform the consumers' ideas into chemical and physical parameters of the final food product. Of course, market research can help a lot when it is combined with profound physicochemical knowledge of the product. This also will help to develop understanding and models (which could be simply data-based) able to link the properties of the product and the characteristics that the final consumer looks for.

The thing here is: are there innovative ways, based on digital tools, to identify consumer needs? Ideally, social networks can reveal some consumer needs and preferences, but to date, there are no studies showing how data on consumer preferences (taken from social networks, or any other virtual space where consumers commonly upload and share food images but also recipes, cooking images or videos, and food diaries, leading to large-scale food data) can be translated in bounded sensorial preferences about a given class of products or a specific product. An interesting review of computing technology applied to the food virtual/social world has been presented by Min et al. (7). There are also studies on possibilities offered by social media in identifying consumers' preferences, and they are discussed in the following paragraphs.

### “Tweet What You Eat”

An interesting example of using social networks to capture some trends about consumers' preferences was provided by Abbar et al. (8). Their starting question was: Can we use social media to get insights into the dietary habits of a community? The authors analyzed the potential of social networks to provide insight into dietary choices of 210K US resident Twitter users by linking the tweeted dining experiences to their interests, demographics, and social networks. It was found out that a correlation exists among the foods mentioned in the daily tweets (of analyzed users), the local obesity and diabetes statistics. Calculating the energy content related to mentioned foods, authors found out how food tweeted energy content is correlated to user interest and demographic indicators, and that users. No doubt that there is a lot to do to develop sensitive and accurate tools for user characterization, with both textual and social network information available. For example, the fact itself that in a place with a high percentage of population suffering from obesity most of the tweets talk about high energy foods does not reveal anything specific on the food preferences of consumers in such a place. The analysis must be crossed with other kinds of information. Or imagine a start-up interested to launch a new plant-based gelato. Before diving into the preferred product characteristics, the start-up maybe wants to target where the ice cream lovers are. Distinguishing foods tweets between rural and urban zones (8) one may conclude that consumers from the rural area are the right target. On the other hand, in large urban areas consumers are more open to adopting a plant-based diet and are more prone to spend some dollars more to buy a healthier product as a plant-based gelato could be.

### Apps and Food

Differently than social networks, apps directly related to the food world may help better in this task. Most of the people thinking about app and food think just about an app for food delivery. But these apps are not the only ones. Many apps about nutrition/diet exist as well as apps about food preparation. Also, apps pretending to help reduce food waste are gaining interest. Some of them cover more than one goal at the same time (9). Let us see if and how they can help with food product design.

Apps on nutrition/diet/ health are sources of data on true food consumption, consumer habits, and dietary requirements: very important data, also going toward possible personalized nutrition.

Franco et al. (10) analyzed the main features of the most popular nutrition apps and compare their strategies and technologies for dietary assessment and user feedback. By exploring stores from main providers of smartphone services, authors found 13 apps using several keywords, such as diet tracker, dietician, eating, fit, fitness, food, food diary, food tracker, health, lose weight, nutrition, nutritionist, weight, weight loss, weight management, weight watcher, and so on. Most of these apps allow to download of the diet diary of active users, thus providing quite detailed information on their daily food intake, in terms of food items, quantity, and – sometimes – preparation. Weber and Achananuparp (11) used the public food diaries of more than 4,000 long-term active users of an app called MyFitnessPal. The heart of their analysis was a classifier able to predict the diet trajectory with respect to a certain self-defined calorie goal, just using the list of foods consumed by a user. Even if the purposes of the above-mentioned pieces of research were other than understanding consumer needs and preferences, there is no doubt that the apps mentioned in these pieces of research are surely a very good source of data to be used to classify consumers' needs and preferences when talking about diet, food products, and their characteristics, at least in terms of calories, composition and nutrition facts.

As an example, it can be interesting to look at the info one can download from the MyFitnessPal database, with daily meals, branded products consumed, also according to the lifestyle of the consumers. Marketing-wise, apps like this are also very useful to push the user to get rewards. And here the healthy characteristics of a branded product (think about a snack) can be used to market that product through the app.

### Food Blogs and AI: Comment – Instruction

A very good source about consumer taste preferences is given by apps about food preparation, where also data about consumer habits and leftovers can be mined. On blogs about food preparation, or reviews about specific food products sold online, we can read the reviews from consumers.

These are good sources of info. It is possible to identify what people like most and, also, why people like a particular food product or preparation. Techniques of word to vectors can be used in such cases. Furthermore, there are artificial intelligence techniques, often using expert knowledge, to relate a comment to an action, instruction, or at least to a suggestion (12).

The readers can have a look at a recipe blog (13) where – in the case of chocolate oatmeal cookies – comments like the following can be found:

“This recipe did not turn out well. Flat and greasy and inedible.”
“We didn't like the combination of chocolate and cinnamon at all….for those that love chocolate and cinnamon together - you'll like this recipe.”
“I saw the picture and was excited to try this recipe! However,… They are not as moist or as sweet as I expected them to be.”

It is not difficult to translate comments like these into recommendations for better food preparation. Being too greasy, the recipe maybe needs a reduction of the fat content or the selection of another fat ingredient. If the final product (the cookie) is found to be too brown, this can suggest reducing the baking temperature or the baking time. If it is too hard, again the baking time or temperature can be reduced, as well as the flour content, or another flour ingredient must be found.

## Digital Tools to Predict Processing Effects on the Final Product

Some interesting examples discussed how algorithms can help even during domestic cooking sessions (14), but undoubtedly at the industrial level is the mechanistic modeling which would help in simulating the effect of ingredients and processing on the final properties of a certain food product (15). Mathematical modeling of transport phenomena is a consolidated science and there is a good choice of software for dealing with it (1). More complicated is the thermodynamics modeling for the prediction of properties, which often needs a combination of heuristics, models based on experience, whereas available data on properties are still scattered and not unified for immediate usage.

**Figure 1:**
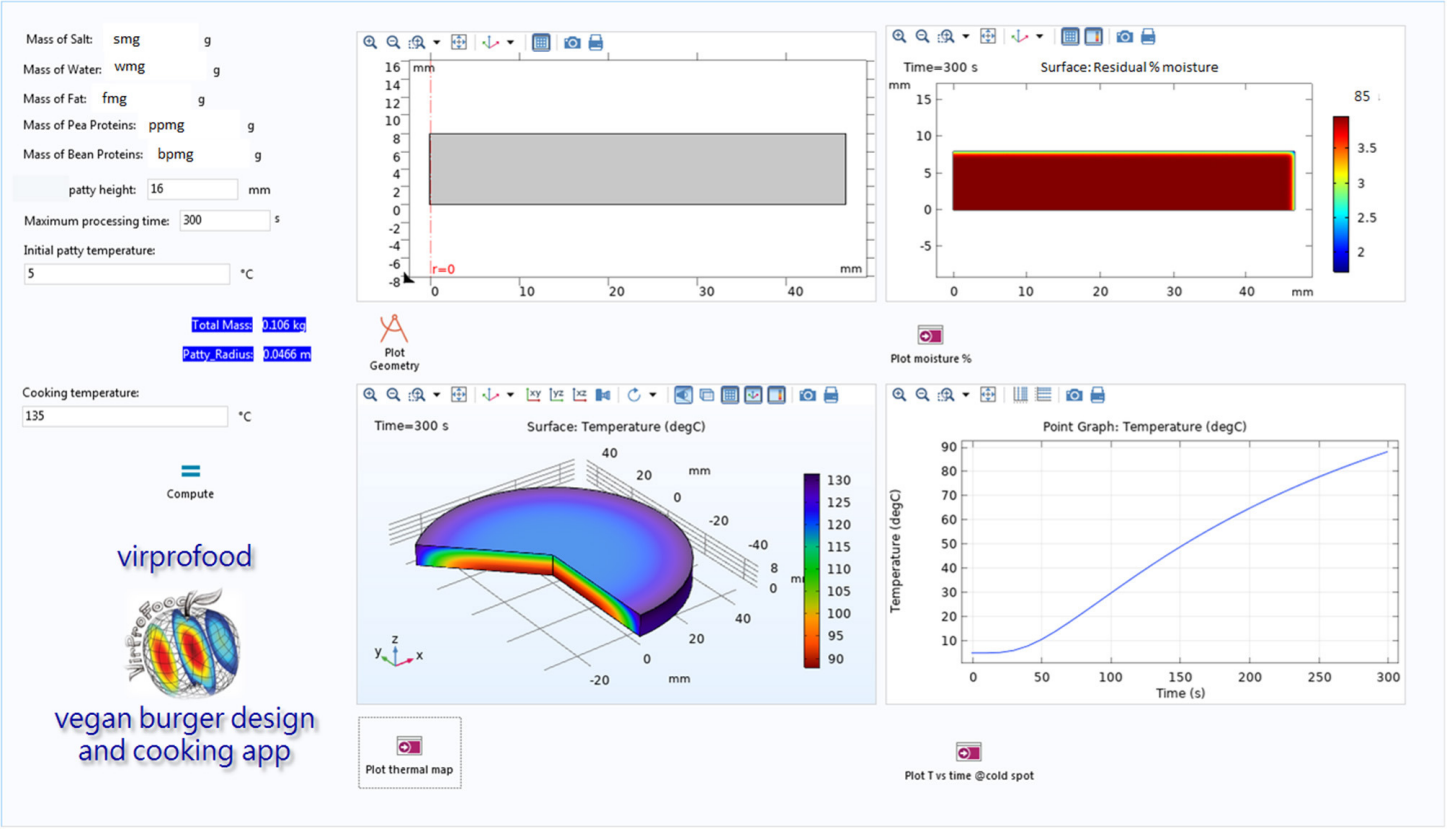
Graphical user interface of an app for the design and the cooking simulation of plant-based burger, developed by VirProFood team at University of Salerno, Italy.

Focusing on the mesoscale process, apps based on mechanistic models can help food scientists to predict the behavior of food products undergoing further process, like in the case of a vegan burger cooking. In Figure 1, it is shown the graphical user interface of an app, designed by the VirProFood team at the University of Salerno (Italy) to simulate the heating of a plant-based burger, for which the composition is given as input, predicting the heating behavior and the change in moisture content of the burger, as a function of the burger dimensions, of the cooking temperature and of the heating time. It is the app itself that calculates the thermo-physical properties of the burger based on its compositions. This kind of app will start to be popular, as high computational level software (able to solve quite complicated systems of the partial differential equation describing the evolution of energy, mass, and properties in a food product) can provide the users with a compiler capable to produce easy-to-use apps (as in the case of COMSOL Compiler 5.6).

## Conclusions

There are, of course, challenges to be faced before a completely digital approach can be used for food product design: probably the quality and the quantity of available data are not still enough to capture trends in consumers' needs and preferences. On the other hand, the work already done about vectorization of words related to comments on recipes or commercial food products can quickly bring to hybrid machines able exploit all the capabilities of digital tools. For instance, hybrid machines, because they will be based on approaches when taking into account consolidated modeling techniques and artificial intelligence. Molecular dynamics and thermodynamics can help to discriminate ingredients and predict properties. Mechanistic models can be used for predicting the properties and performances of the product. Artificial Intelligence (machine learning in this case) techniques can be appropriated for relating product features to sensorial attributes preferred by the consumer and also to choose ingredients (12).

No doubt then that the virtual/digital tools can accelerate time to market for new food products. The exploitation of these tools needs a multidisciplinary eco-system, where food engineers, food scientists, and experts in ingredients and nutrition will seat together with computer scientists to find new ways.

## Data Availability Statement

The original contributions presented in the study are included in the article/supplementary material, further inquiries can be directed to the corresponding author/s.

## Author Contributions

The author confirms being the sole contributor of this work and has approved it for publication.

## Conflict of Interest

The author declares that the research was conducted in the absence of any commercial or financial relationships that could be construed as a potential conflict of interest.

## Publisher's Note

All claims expressed in this article are solely those of the authors and do not necessarily represent those of their affiliated organizations, or those of the publisher, the editors and the reviewers. Any product that may be evaluated in this article, or claim that may be made by its manufacturer, is not guaranteed or endorsed by the publisher.
